# Supplementary material for: Investigating the causal effect of maternal vitamin B12 and folate levels on offspring birthweight
Source: Int J Epidemiol. 2020 Dec 21;50(1):179–89. doi: 10.1093/ije/dyaa256 (PMC7938507; doi:10.1093/ije/dyaa256)
Supplement: dyaa256_Supplementary_Data [file dyaa256_supplementary_data.zip › ije-2020-04-0618-File005.docx]

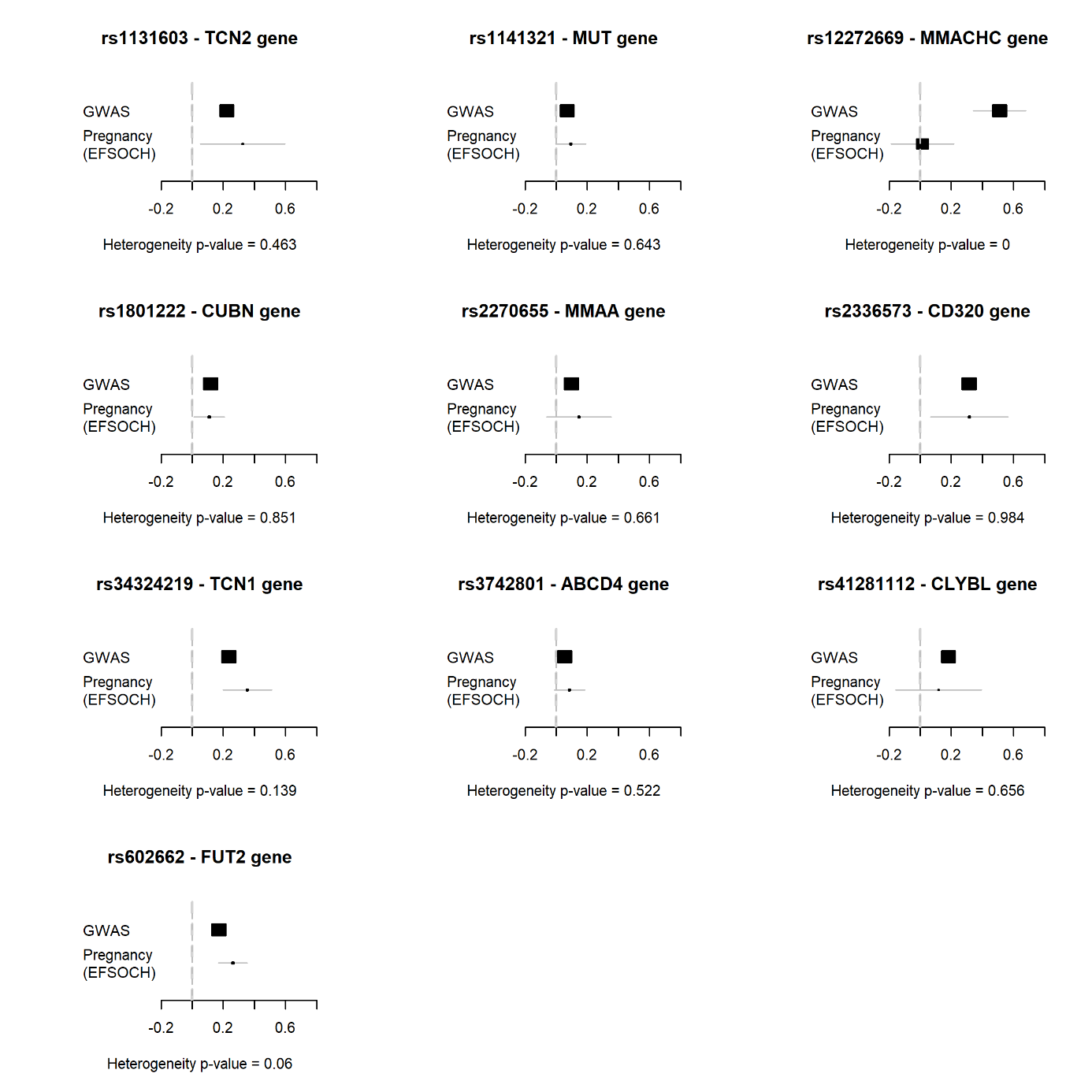


**Supplementary Figure** **S1: Forest plot comparing the SNP effect sizes on B12 from the exposure GWAS used in the MR analysis with the corresponding SNP effects on B12 measured in pregnant women from the EFSOCH study.** All plots show overlapping confidence intervals and no sign of heterogeneity except for in the rs12272669 variant in the *MMACHC* gene (heterogeneity p = 0.0002)*. *We used a proxy for this variant in the EFSOCH study, rs11234541, which was in high LD with rs12272669 but had low imputation quality. SNP: Single Nucleotide Polymorphism; GWAS: Genome-Wide Association Study; MR: Mendelian Randomization; EFSOCH: The Exeter Family Study of Childhood Health; LD: Linkage Disequilibrium.


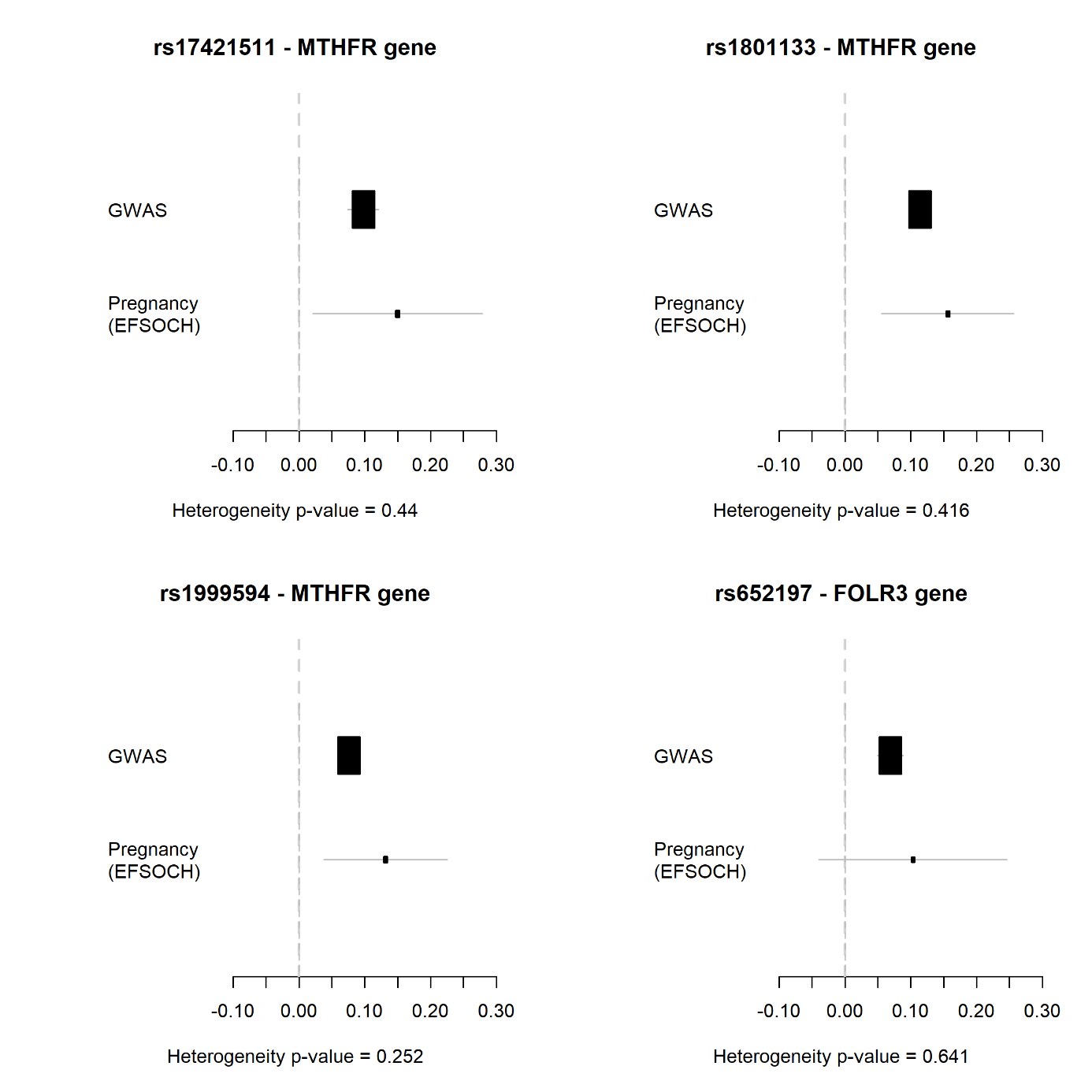


**Supplementary Figure** **S2: Forest plot comparing the SNP effect sizes on folate from the exposure GWAS used in the MR analysis with the corresponding SNP effects on folate measured in pregnant women from the EFSOCH study.** All plots show overlapping confidence intervals and no sign of heterogeneity. SNP: Single Nucleotide Polymorphism; GWAS: Genome-Wide Association Study; MR: Mendelian Randomization; EFSOCH: The Exeter Family Study of Childhood Health.


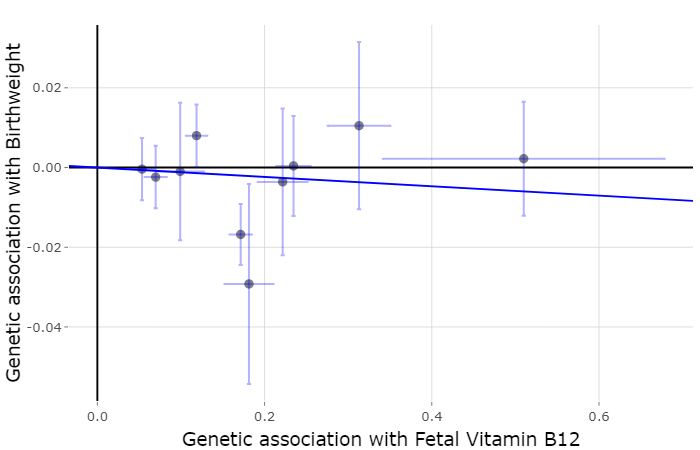


**Supplementary Figure** **S3:** **Mendelian randomization estimates of the causal effect of fetal vitamin B12 levels on offspring birthweight.** X-axis shows the Single Nucleotide Polymorphism (SNP) effect, and standard error, on vitamin B12 levels for each of the ten SNPs and Y-axis shows the SNP effect, and standard error, on offspring birthweight. The regression line for the Inverse variance weighted Mendelian Randomization method is shown.


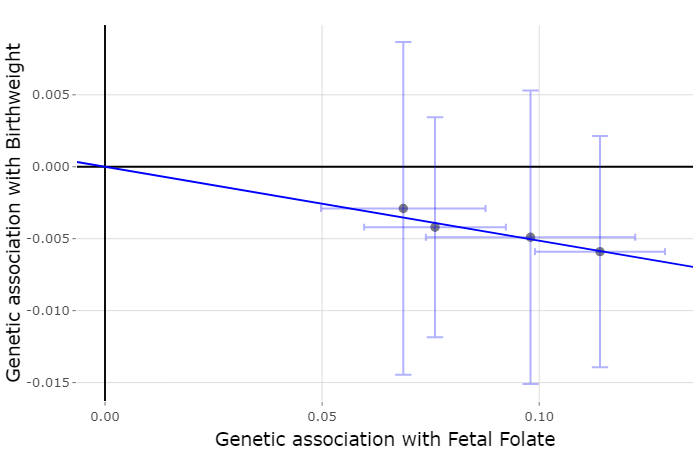


**Supplementary Figure** **S4:** **Mendelian randomization estimates of the causal effect of fetal folate levels on offspring birthweight.** X-axis shows the Single Nucleotide Polymorphism (SNP) effect, and standard error, on folate levels for each of the four SNPs and Y-axis shows the SNP effect, and standard error, on offspring birthweight. The regression line for the Inverse variance weighted Mendelian Randomization method is shown.
